# Polymorphisms of the *XRCC1*, *XRCC3*, &* XPD *genes, and colorectal cancer risk: a case-control study in Taiwan

**DOI:** 10.1186/1471-2407-5-12

**Published:** 2005-01-28

**Authors:** Chih-Ching Yeh, Fung-Chang Sung, Reiping Tang, Chung Rong Chang-Chieh, Ling-Ling Hsieh

**Affiliations:** 1 Institute of Environmental Health, National Taiwan University College of Public Health, Taipei 100, Taiwan; 2 Institute of Environmental Health and Department of Risk Management, China Medical University College of Public Health, Taichung 404, Taiwan; 3 Colorectal Section, Chang Gung Memorial Hospital, Linkou, Taiwan; 4 Department of Public Health, Chang Gung University, Tao-Yuan 333, Taiwan

## Abstract

**Background:**

Recent studies relating to the association between DNA repair-gene polymorphisms and colorectal cancer risk would, to the best of our knowledge, appear to be very limited. This study was designed to examine the polymorphisms associated with three DNA repair genes, namely: *XRCC1 *Arg399Gln, *XRCC3 *Thr241Met and *XPD *Lys751Gln, and investigate their role as susceptibility markers for colorectal cancer.

**Methods:**

We conducted a case-control study including 727 cases of cancer and 736 hospital-based age- and sex-matched healthy controls to examine the role of genetic polymorphisms of three DNA-repair genes (*XRCC1*, *XRCC3 *and *XPD*) in the context of colorectal cancer risk for the Taiwanese population. Genomic DNA isolated from 10 ml whole blood was used to genotype *XRCC1 *Arg399Gln, *XRCC3 *Thr241Met and *XPD *Lys751Gln by means of polymerase chain reaction (PCR) and restriction fragment length polymorphism (RFLP) analysis.

**Results:**

The risk for colorectal cancer did not appear to differ significantly amongst individuals featuring the *XRCC1 *399Arg/Arg genotype (OR = 1.18; 95% CI, 0.96–1.45), the *XRCC3 *241Thr/Thr genotype (OR = 1.25; 95% CI, 0.88–1.79) or the *XPD *751Gln allele (OR = 1.20; 95% CI, 0.90–1.61), although individuals featuring a greater number of risk genotypes (genotype with OR greater than 1) did experience a higher risk for colorectal cancer when compared to those who didn't feature any risk genotypes (Trend test *P *= 0.03). Compared with those individuals who didn't express any putative risk genotypes, individuals featuring all of the putative risk genotypes did experience a significantly greater cancer risk (OR = 2.43, 95% CI = 1.21–4.90), particularly for individuals suffering tumors located in the rectum (OR = 3.18, 95% CI = 1.29–7.82) and diagnosed prior to the age of 60 years (OR = 4.90, 95% CI = 1.72–14.0).

**Conclusions:**

Our results suggest that DNA-repair pathways may simultaneously modulate the risk of colorectal cancer for the Taiwanese population, and, particularly for rectal cancer and younger patients.

## Background

Humans are routinely exposed to mutagenic and carcinogenic aromatic amines via smoking, well-cooked food and other sources [1]. These chemicals can form DNA adducts *in vivo *and thus lead to DNA damage [2]. The integrity of most of the so-damaged DNAs is typically restored as a consequence of the action of certain DNA-repairing enzymes, the normal function of which is important for maintaining genomic integrity and preventing cellular neoplastic transformation [3]. Since the genetic polymorphisms of DNA-repair enzymes might be able to influence DNA adduct levels [4-6], the particular degree of DNA-repair capacity has often been associated with the risk of human cancers [7-11].

Amongst the known genetic polymorphisms of the DNA-repair genes [12], the xeroderma pigmentosum group D (XPD, also known as ERCC2) and x-ray repair cross-complementing groups 1 and 3 (XRCC1 and XRCC3) have been studied most commonly [13]. The *XPD *gene encodes a helicase that is a component of the transcription factor TFIIH [14], this factor being an essential member of the nucleotide-excision repair (NER) pathway that is responsible for effecting repairs to bulky adducts and UV-induced DNA damage [15]. In 2002, Qiao et al. [16] reported that individuals featuring *XPD *751Gln/Gln did demonstrate suboptimal DNA-repair capacity (DRC) in regard to its ability to remove UV photoproducts when compared to the *XPD *751Lys/Lys and Lys/Gln genotypes. The XRCC1 protein is a scaffolding protein directly associated with polymerase beta, DNA ligase III and poly (ADP-ribose) polymerase (PARP) and functions in a complex to facilitate the base-excision repair (BER) and single-strand break-repair processes [17-19]. In 1999, Lunn et al. noted that individuals harboring the *XRCC1 *399Gln allele were associated, more significantly, with higher levels of both aflatoxin B1-DNA adducts and glycophorin A variants when compared to individuals who exhibited the Arg/Arg genotype [4]. XRCC3 participates in DNA double-strand break repair and is a member of an emerging family of Rad-51-related proteins that likely participate in homologous recombinational repair (HRR) in order to maintain chromosome stability [20].

To the best of our knowledge, studies pertaining to these DNA-repair genes focusing on colorectal cancer risk would appear to be limited and controversial. In 2000, Abdel-Rahman et al. [21] observed that the *XRCC1 *399Gln allele, compared to the *XRCC1 *399Arg/Arg genotype, was associated with an increased risk for developing colorectal cancer, especially amongst young urban residents, although in 2003, Mort et al. [22] failed to reveal any significant associations between colorectal cancer risk and any polymorphisms of four of the NER genes (*XPD*, *XPF*, *XPG*, *ERCC1*) or *XRCC1*. In the present paper, we conducted a hospital-based case-controlled study to examine the role of genetic polymorphisms of three DNA-repair genes (*XRCC1*, *XRCC3 *and *XPD*) in the context of colorectal cancer risk for the Taiwanese population.

## Methods

### Subjects

Detailed descriptions of the specific characteristics of the study participants have been published previously [23]. In brief, participants were recruited from the Chang Gung Memorial Hospital between January 1995 and January 1999 inclusively. The colorectal adenocarcinoma cancer cases (*n *= 776) participating in this study were newly diagnosed and histologically confirmed. Patients suffering from familial adenomatous polyposis, hereditary nonpolyposis colorectal cancer, or inflammatory bowel disease and other related malignancies were excluded from study participation. Seven hundred and twenty-seven of the original 776 cases (94%) were finally included in this study. Seven hundred and forty-seven age (same age) and sex-matched controls were recruited from the Physical Check-Up Department during the same period. All the participating controls had received comprehensive health examinations including colonoscopies. After excluding individuals diagnosed with other colorectal diseases, a history of other cancers or the existence of a family history of colorectal cancer, 736 controls (98%, 736/747) were finally included in this study. With informed consent, the socio-demographic characteristics of study participants were ascertained by means of the application of a structured questionnaire, at which time 10 ml of venous blood was collected.

### Genotyping

Genomic DNA isolated from 10 ml whole blood was used to genotype *XRCC1 *Arg399Gln, *XRCC3 *Thr241Met and *XPD *Lys751Gln by means of polymerase chain reaction (PCR) and restriction fragment length polymorphism (RFLP) analysis. All of the PCR reactions were carried out by a Mastercycler gradient thermocycler (Eppendorf, Hamburg, Germany) in a final volume of 25 ul containing 200 ng of each primer, 50 ng genomic DNA, 1.5 mM MgCl_2_, 200 ul dNTPs and 1.0 unit of Taq DNA Polymerase in the buffer provided by the manufacturer. In addition, all laboratory genotyping personnel were blind to the case-control status of the samples.

The 615 bp *XRCC1 *PCR products were amplified with the primers 5'-TTGTGCTTTCTCTGTGTCCA-3' (sense) and 5'-TCCTCCAGCCTTTTCTGATA-3' (antisense) and digested with *Msp*I (New England BioLabs, Beverly, MA, USA). The Arg allele revealed 374 and 221 bp fragments following digestion and polyacrylamide gel electrophoresis, whilst the Gln allele was not digested by *Msp*I [4]. The 136 bp *XRCC3 *PCR products were amplified with the primers 5'-GCCTGGTGGTCATCGACTC-3' (sense) and 5'-ACAGGGCTCTGGAAGGCACTGCTCAGCTCACGCACC-3' (antisense) and digested with *Nco*I (New England BioLabs). The Met allele revealed 97 and 39 bp fragments following digestion and polyacrylamide gel electrophoresis, while the Thr allele was not digested by *Nco*I [24]. The 734 bp *XPD *PCR products were amplified with the primers 5'-CCTCTCCCTTTCCTCTGTTC-3' (sense) and 5'-CAGGTGAGGGGGACATCT-3' (antisense) and digested with *Pst*I (Takara, Japan). The Gln allele revealed 646 and 88 bp fragments following digestion and polyacrylamide gel electrophoresis, whilst the Lys allele was not able to be digested by *Pst*I [25].

### Statistical analysis

Tests for Hardy-Weinberg equilibrium amongst controls were conducted using observed genotype frequencies and a chi-square test featuring one degree of freedom. Baseline sociodemographic characteristics between cases and controls were analyzed using the chi-square test and two-sample Students' *t*-test. Multivariate unconditional logistic regressions were used to examine the association between the *XRCC1*, *XRCC3 *and *XPD *genotypes and the risk for colorectal cancer. Since the differences in the estimated risks between conditional logistical regression and unconditional logistical regression were small, unconditional logistical regression was used to estimate odds ratio (OR) and 95% confidence interval (CI) with the matching factors (age and gender) included in the model for estimation [26]. Other potential confounders (such as physical activity, cigarette smoking, alcohol use, coffee intake, and consumption of staple, meat, vegetable/fruit and fish/shrimp) would be also included if they have >10% effect on the gene main effects. However, none of these factors have met the inclusion criteria.

Based on the multiplicative scale, the likelihood ratio test was further used to evaluate the interaction between *XRCC1*, *XRCC3 *and *XPD *genes on the risk for the colorectal cancer. Since the associations between polymorphisms of DNA-repair gene, cancer susceptibility and DNA repair capacity are inconsistent [13], we defined the risk allele as the allele with OR>1 observed in the present study. Stratified analyses were also conducted to evaluate the differences between specific tumor sites (colon and rectum) and age groups (< = 60 years old and > 60 years old). All analyses were performed using the SAS statistical package (version 8.1 for windows; SAS Institute, Inc., Cary, NC, USA) and all tests were two-sided.

## Results

More men (56%) than women participated in this study (Table 1). The mean age for both groups was 60 years. From the cancer cases, 352 patients suffered from colon cancer (48%) and 375 patients from rectal cancer (52%), with most such cases being deemed to be at stage II or stage III (both were 33%). Detailed analyses of the sociodemographic characteristics and potential risk factors associated with colorectal cancer amongst the study population have been published previously [23].

The genotypic distributions of the three DNA-repair genes for both cancer cases and controls are shown in Table 2. The frequencies for the *XRCC1 *399Gln, *XRCC3 *241Met and *XPD *751Gln allele amongst the controls were, respectively, 0.27, 0.05 and 0.07, these genotype frequencies being in Hardy-Weinberg equilibrium. Due to the relatively low frequencies of variant alleles for these genes in the present study, any genotype featuring one or more variant alleles was combined for further analyses. The risk for colorectal cancer was not significantly different for individuals featuring the *XRCC1 *399Arg/Arg genotype (OR = 1.18; 95% CI, 0.96–1.45), the *XRCC3 *241Thr/Thr genotype (OR = 1.25; 95% CI, 0.88–1.79) or the *XPD *751Gln allele (OR = 1.20; 95% CI, 0.90–1.61).

As revealed in Table 3, those individuals exhibiting a greater number of risk genotypes (genotype with OR greater than 1) faced a greater risk for colorectal cancer when compared to those individuals who did not display any risk genotypes (Trend test, *P *= 0.03). Subjects who demonstrated two or three of the putative risk genotypes did reveal a significantly greater risk for colorectal cancer (OR = 1.95; 95% CI, 1.08–3.52 and OR = 2.43; 95% CI, 1.21–4.90, respectively) as compared to those individuals who did not feature any putative risk genotypes, although it appears that no gene-gene interactions arose amongst these three genes (all *P *levels for interaction were >0.21). When stratified by tumor site and age at diagnosis, these combined gene effects upon cancer risk were observed for individuals who revealed that their tumor was located in the rectum (Trend test *P *= 0.03) and those individuals for whom their tumor was diagnosed prior to their being 60 years of age (Trend test *P *= 0.004; Table 4). The ORs for subjects with three putative risk genotypes were 3.18 (95% CI, 1.29–7.82) for rectal cancer and 4.90 (95% CI, 1.72–14.0) for those individuals diagnosed prior to 60 years of age, respectively.

## Discussion

To the best of our knowledge, few studies have investigated the role of polymorphisms in DNA-repair genes for patients suffering colorectal cancer [21,22]. In this hospital-based case-control study of colorectal cancer-suffering patients in Taiwan, we found polymorphisms in three DNA-repair genes associated with an elevated risk of colorectal cancer. In addition, a gene-dosage effect was found for rectal cancer and younger patients. These findings suggest that those genes involved in different DNA-repair pathways may act simultaneously in the process of carcinogenesis for colorectal cancer.

Although we didn't find any significant independent associations between these DNA-repair genes and colorectal cancer risk, the risk appeared to be slightly increased for individuals who featured the *XRCC1 *399Arg/Arg, *XRCC3 *241Thr/Thr genotypes and the *XPD *751Gln allele. Our results do not appear to be entirely consistent with the results of some previous reports [21,22], the former group reporting that the *XRCC1 *399Gln allele significantly increased the risk of colorectal cancer (OR = 3.98, 95% CI = 1.50–10.6). In 2003, Mort et al. [22] noted that the risk of suffering colorectal cancer was significantly heightened for individuals who featured the *XRCC3 *241Thr allele (OR = 1.52, 95% CI = 1.04–2.22) and only slightly increased for those individuals who revealed the *XRCC1 *399Gln and *XPD *751Gln alleles. In 2001, Park et al. [27] reported that advanced colorectal cancer-suffering patients who revealed the *XPD *751Gln/Gln genotype featured a poorer (positive) response rate to chemotherapy and also a shorter survival period compared with colorectal cancer-suffering individuals who belonged to either the 751Lys/Lys or the 751Lys/Gln group.

The apparent divergence between these studies and ours might be due to one of two reasons. Firstly, it may simply be that ethnic differences in allele frequency for the polymorphism might explain the controversial findings. The frequencies for the *XRCC1 *399Gln, *XRCC3 *241Met and *XPD *751Gln alleles amongst the healthy controls in this study (0.27, 0.05 and 0.07, respectively) were similar to the results for other studies conducted in Taiwan [28,29] and China [30,31], but appeared to be much lower than those reported in 2003 by Mort et al. [22] (0.42 for *XRCC1 *399Gln, 0.45 for *XRCC3 *241Met and 0.36 for *XPD *751Gln) for a British population. On the other hand, the *XRCC1 *399Gln allele frequency observed in the present study is greater than that observed by Abdel-Rahman et al. in 2000 (0.14 for *XRCC1 *399Gln) [21]. Abdel-Rahman et al. also found that urban residents have 9.97-fold increased risk of early-onset colorectal carcinoma than rural residents with the *XRCC1 *399Gln allele [21]. Therefore, it is possible that the divergence in results from different studies might be related to different levels of carcinogen exposure for different populations. Moreover, inadequate study design such as a too-small sample size and/or the inadequate controlling for certain confounders (such as age and gender) should also be considered as constituting the underpinning for such differing results.

Combined effects of polymorphisms of the *XRCC1 *Arg399Gln, *XRCC3 *Thr241Met and *XPD *Lys751Gln genes in regard to colorectal cancer risk were observed in the present study. With the complexity of detail of environmental exposures to various carcinogens, it is plausible that the effective repair of DNA damaged by chemical mixtures necessitates multiple DNA-repair pathways (including BER, HRR and NER pathways). The failure of, or the presence of deficient DNA repair capacity for each specific DNA-repair pathway may contribute to an increase in cancer risk. Additive or multiplicative effects of combined genetic variants for different DNA-repair pathways have been previously reported for lung cancer [32], melanoma [33] and breast cancer [34]. In 2003, Zhou et al. [35] found that the risk of lung cancer amongst nonsmokers increased progressively with the increase in the number of high-risk alleles of *XRCC1 *and *XPD *genes. Hu et al. [36] also observed that prolonged cell-cycle delay was significantly associated with the number of variant alleles of the *APE1 *and *XRCC1 *genes that were present. It would therefore appear reasonable to hypothesize that genetic polymorphism(s) for DNA-repair genes may simultaneously contribute to colorectal cancer susceptibility.

In this study, we found that the combined effect of multiple DNA-repair genes upon colorectal cancer risk was significant for our younger age group, but not so for the older age group. This finding appears to be similar to the results of most of the previous studies pertaining to colorectal cancer [21], basal-cell carcinoma [37], head-and-neck cancer [38], hepatocellular carcinoma [39], and lung cancer [6,32,35,40] that we reviewed, although we did note that one study reported that such an elevated risk was also observed for old-aged head-and-neck cancer patients [41]. In addition, few studies that we reviewed failed to find a difference between age groups in regard to for cancer risk [31,42-46]. In 2000, Duell et al. [47] noted that old healthy subjects who featured the *XRCC1 *399Gln allele appeared to be significantly associated with detectable DNA adducts in their blood mononuclear cells when compared to younger subjects who featured the 399Arg/Arg genotype. In 2001, Hemminki et al. [48] also found that old subjects who revealed the *XPD *751Gln/Gln genotype exhibited a decreased DNA-repair rate for, specifically, UV-specific cyclobutane pyrimidine dimers in the skin. Therefore, it is possible that individuals who demonstrate a less-efficient DNA-repair capacity might develop tumors at a younger age than individuals who reveal an efficient DNA-repair capacity.

In our study, we also found that the combined genetic effect of these three DNA-repair genes in regard to cancer risk was more pronounced for the rectum than for the colon. This difference may reflect certain etiological differences between colon and rectal carcinogenesis. In fact, different epidemiological characteristics, etiology, pathogenesis and clinical behavior have been reported for different anatomical sites of colorectal cancer [49,50], this latter group suggesting that carcinogenesis within the distal colon was associated with bulky-adduct-forming (BAF) agents and that these DNA lesions were repaired through NER pathways. In 2001, Hong et al. [51] also found that much more DNA-repair and apoptosis activity occurred in the distal rather than the proximal colon for the rat azoxymethane carcinogenesis model. Taken together, these observations support the notion that insufficient DNA-repair capacity could contribute to the risk of cancer associated with exogenous carcinogen exposure.

To the best of our knowledge, this study is the first to report on *XRCC1*, *XRCC3 *and *XPD *polymorphisms in relation to the risk of colorectal cancer for the Taiwanese population. Our results suggest that genetic polymorphisms of the *XRCC1*, *XRCC3 *and *XPD *genes, particularly in combination, may be associated with an individual's susceptibility to colorectal cancer. Acknowledging the relatively limited sample size in the subgroups for the low allelic frequencies, further studies incorporating a larger sample size and/or another ethnic population are needed to confirm the genetic role of DNA-repair mechanisms as regards colorectal cancer susceptibility.

## Conclusions

Our results suggest that DNA-repair pathways may simultaneously modulate the risk of colorectal cancer for the Taiwanese population, and, particularly for rectal cancer and younger patients.

## Competing interests

The author(s) declare that they have no competing interests.

## Authors' contributions

CCY carried out the genotyping analysis, performed the statistical analysis and drafted the manuscript. FCS participated in the design of the study. RT and CRCC participated in the design of the study and provided clinical biospecimens. LLH conceived of the study, and participated in its design and coordination. All authors read and approved the final manuscript.

## Pre-publication history

The pre-publication history for this paper can be accessed here:



## Figures and Tables

**Table 1:** Characteristic details for the cases and controls

Variables	Cases, n (%) (N = 727)	Control, n (%) (N = 736)	p
Gender			0.75^a^
Male	410 (0.56)	409 (0.56)	
Female	317 (0.44)	327 (0.44)	
Mean age (SD), years	60.3 (12.8)	60.7 (13.0)	0.64^b^
Colon/rectum, N	352/375	-	
Stage			
0	9 (1.2)	-	
I	84 (11.6)	-	
II	242 (33.3)	-	
III	241 (33.2)	-	
IV	151 (20.8)	-	

a: Chi-square test

b: t test

**Table 2:** Distribution of three DNA repair genes for cases and controls

Polymorphism	Cases, n (%)	Control, n (%)	OR (95% CI) ^a^
XRCC1 Arg399Gln			
Arg/Arg	407 (56.7)	384 (52.7)	1.00
Arg/Gln	260 (36.2)	291 (39.9)	0.84 (0.67–1.07)
Gln/Gln	51 (7.1)	54 (7.4)	0.82 (0.53–1.25)
Missing	9	7	
With Gln^b^	311 (43.3)	345 (47.3)	1.00
Arg/Arg	407 (56.7)	384 (52.7)	1.18 (0.96–1.45)
			
XRCC3 Thr241Met			
Thr/Thr	660 (91.5)	658 (89.7)	1.00
Thr/Met	60 (8.3)	74 (10.1)	0.83 (0.56–1.21)
Met/Met	1 (0.1)	2 (0.3)	0.50 (0.05–5.51)
Missing	6	2	
With Met^c^	61 (8.5)	76 (10.4)	1.00
Thr/Thr	660 (91.5)	658 (89.7)	1.25 (0.88–1.79)
			
XPD Lys751Gln			
Lys/Lys	602 (84.0)	631 (86.3)	1.00
Lys/Gln	112 (15.6)	96 (13.1)	1.29 (0.94–1.76)
Gln/Gln	3 (0.4)	4 (0.6)	0.81 (0.18–3.62)
Missing	10	5	
Lys/Lys	602 (84.0)	631 (86.3)	1.00
With Gln^d^	115 (16.0)	100 (13.7)	1.20 (0.90–1.61)

a: ORs and 95% CIs were estimated from unconditional logistic regressions, controlling for age and gender

b: Arg/Gln+ Gln/Gln

c: Thr/Met+ Met/Met

d: Lys/Gln+ Gln/Gln

**Table 3:** Combined effect of *XRCC1*, *XRCC3 *and *XPD *genes upon colorectal cancer risk

XRCC1 Arg399Gln	XRCC3 Thr241Met	XPD Lys751Gln	Cases	Controls	OR (95% CI)^a^	OR (95% CI)^a^
With Gln	With Met	Lys/Lys	18	34	1.00	1.00
Arg/Arg	With Met	Lys/Lys	30	30	1.91 (0.89–4.10)	
With Gln	Thr/Thr	Lys/Lys	239	257	1.77 (0.97–3.22)	1.79 (0.99–3.24)
With Gln	With Met	With Gln	6	6	1.90 (0.54–6.76)	
Arg/Arg	Thr/Thr	Lys/Lys	312	307	1.93 (1.07–3.50)	
Arg/Arg	With Met	With Gln	7	5	2.65 (0.74–9.56)	1.95 (1.08–3.52)
With Gln	Thr/Thr	With Gln	48	46	1.99 (0.99–4.01)	
Arg/Arg	Thr/Thr	With Gln	54	42	2.43 (1.21–4.90)	2.43 (1.21–4.90) 0.03^b^

a: ORs and 95% CIs were estimated from unconditional logistic regressions, controlling for age and gender

b: Trend test

**Table 4:** Number of risk-genotypes of three DNA repair genes and colorectal cancer risk by age at diagnosis and by tumor site

	Number of risk-genotypes*				
		
	0	1	2	3	P^b^
Age at diagnosis					
< = 60 years					
Cases/Controls	7/20	114/126	169/155	29/17	
OR	1.0	2.58	3.12	4.90	0.004
(95% CI)^a^	(reference)	(1.05–6.33)	(1.28–7.58)	(1.72–14.0)	
>60 years					
Cases/Controls	11/14	161/167	198/203	25/25	
OR	1.0	1.24	1.25	1.28	0.74
(95% CI)^a^	(reference)	(0.55–2.82)	(0.55–2.83)	(0.49–3.35)	
Tumor site					
Colon					
Cases/Controls	10/34	132/293	177/358	23/42	
OR	1.0	1.51	1.65	1.86	0.16
(95% CI)^a^	(reference)	(0.72–3.14)	(0.80–3.42)	(0.78–4.44)	
Rectum					
Cases/Controls	8/34	143/293	190/358	31/42	
OR	1.0	2.13	2.32	3.18	0.03
(95% CI)^a^	(reference)	(0.96–4.73)	(1.05–5.11)	(1.29–7.82)	

*Risk-genotype was defined as genotype with OR>1 as shown in Table 3.

a: ORs and 95% CIs were estimated from unconditional logistic regressions, controlling for age and gender

b: p for trend test
